# Microglial cell response in α7 nicotinic acetylcholine receptor-deficient mice after systemic infection with *Escherichia coli*

**DOI:** 10.1186/s12974-022-02452-8

**Published:** 2022-04-12

**Authors:** Inge C. M. Hoogland, Jutka Yik, Dunja Westhoff, Joo-Yeon Engelen-Lee, Merche Valls Seron, Wing Kit Man, Judith H. P. M. Houben-Weerts, Michael W. T. Tanck, David J. van Westerloo, Tom van der Poll, Willem A. van Gool, Diederik van de Beek

**Affiliations:** 1 Department of Neurology, Amsterdam University Medical Centres, Location Academic Medical Center, Amsterdam Neuroscience, University of Amsterdam, PO Box 22660, 1100DD Amsterdam, The Netherlands; 2 Centre of Experimental Molecular Medicine, Amsterdam University Medical Centres, Location Academic Medical Center, University of Amsterdam, Amsterdam, The Netherlands; 3 Department of Clinical Epidemiology, Amsterdam University Medical Centres, Location Academic Medical Center, University of Amsterdam, Amsterdam, The Netherlands; 4 Intensive Care Medicine, Leiden University Medical Center, Leiden, The Netherlands

**Keywords:** Microglia, Microglial activation, Systemic infection, *Escherichia coli*, Neuro-inflammation, α7 acetylcholine receptor, α7nAChR, Knock-out, Mouse model

## Abstract

**Background:**

Development of neurodegeneration in older people has been associated with microglial cell activation triggered by systemic infection. We hypothesize that α7 nicotinic acetylcholine receptor (α7nAChR) plays an important role in regulation of this process.

**Methods:**

8- to 10-week-old male wild-type (WT) and α7nAChR knock-out (*α7nAChR*^−/−^) mice were intraperitoneally inoculated with live *Escherichia* (*E.*) *coli* or saline. After inoculation, all mice were treated with ceftriaxone (an antimicrobial drug) at 12 and 24 h and killed at 2 or 3 days. The microglial response was characterized by immunohistochemical staining with an ionized calcium-binding adaptor molecule 1 (Iba-1) antibody and flow cytometry. To quantify inflammatory response, mRNA expression of pro- and anti-inflammatory mediators was measured in brain and spleen.

**Results:**

We observed no differences in Iba-1 positive cell number or morphology and flow cytometry (CD11b, CD45 and CD14) of microglial cells between WT and *α7nAChR*^−/−^ mice after systemic infection. Infected *α7nAChR*^−/−^ mice showed significantly higher mRNA expression in brain for tumor necrosis factor alpha (TNF-α) at day 2 and 3, interleukin 6 (IL-6) at day 2 and monocyte chemotactic protein 1 (MCP-1) and suppressor of cytokine signaling 1 (SOCS1) at day 3, there was significantly lower mRNA expression in brain for mitogen-activated protein kinase 1 (MAPK1) at day 2 and 3, high-mobility group 1 (HMGB-1) and CD11b at day 2, and deubiquitinase protein A20 (A20) at day 3 compared to infected WT mice.

**Interpretation:**

Loss of function of α7nAChR during systemic infection led to an increased expression of TNF-α and IL-6 in brain after systemic infection with *E. coli*, but not to distinct differences in microglial cell number or morphological activation of microglia.

**Supplementary Information:**

The online version contains supplementary material available at 10.1186/s12974-022-02452-8.

## Introduction

Delirium has been associated with development of degenerative diseases of the central nervous system (CNS) [1]. It has been hypothesized that systemic inflammation may trigger delirium and subsequently may lead to chronic neuro-inflammation, a process in which microglial cell activation may be key [1, 2]. Impaired cholinergic inhibition of activated microglia in elderly people may contribute to such chronic neuro-inflammation [3].

It has been shown that acetylcholine (ACh), the main parasympathetic neurotransmitter, effectively deactivates peripheral macrophages and inhibits the release of pro-inflammatory mediators [4, 5]. The ACh-dependent macrophage deactivation is mediated by the α7 subunit of the nicotinic ACh receptor (α7nAChR), and has been described as essential for the cholinergic anti-inflammatory pathway [5, 6]. Cholinergic pathways are impaired in several neurodegenerative diseases, like Alzheimer’s and Parkinson’s disease [7]. The α7nAChR is present on microglial cells and the neurotoxic effect of lipopolysaccharide (LPS) activated microglia due to inflammatory cytokines was attenuated by pretreatment of microglia with nicotine in vitro [8]. This suggests an important anti-inflammatory role for α7nAChRs in microglia during inflammation, representing a possible therapeutic target in neuro-inflammatory diseases.

The aim of this study was to investigate the effect of α7nAChRs on microglial cells following a systemic infection. For this we compared microglial activation and inflammatory response in the brain of wild-type (WT) mice with α7nAChR knock-out (*α7nAChR*^*−/−*^) mice after intraperitoneally injection with live *Escherichia* (*E.*) *coli*. Previously we suggested that this infection model simulates the clinical situation closely and therefore is more suited than inoculation with LPS [9].

## Materials and methods

### Animals

In total, 57 specific pathogen-free 8- to 10-week-old male WT C57BL/6 mice were purchased from Charles River (Maastricht, The Netherlands). 57 specific pathogen-free 8- to 10-week-old male *α7nAChR*^*−/−*^ (B6.129S7-Chrna7tm1Bay/J) [10] mice were kindly provided by the Department of Endocrinology and Metabolic disease of Leiden University Medical Center (LUMC). Mice were housed in groups of 2 to 6, in individually ventilated cages (IVC), for at least 2 weeks before testing and maintained at the animal care facility of the Academic Medical Centre (University of Amsterdam). Ambient temperature was 19–24 °C with 40–70% humidity. According to national guidelines, food and water were available ad libitum and a 12:12 h light–dark cycle was retained. All experiments were approved by the Institutional Animal Care and Use Committee of the Academic Medical Centre Amsterdam, the Netherlands (Approval number: DIX102895).

### Bacteria

*Escherichia coli* (K1:O18) were cultured in Luria–Bertani medium (LB) at 37 °C to midlog phase in 1 h and 45 min. The amount of bacteria in the culture was estimated by measuring the *A*600 in a spectrophotometer. *E. coli* were harvested by centrifugation at 3000 rpm for 10 min and washed twice with pyrogen-free sterile isotonic saline. Bacteria were diluted to a final concentration of 1 × 10^4^ colony-forming units (CFU’s)/200 μl. Serial tenfold dilutions of the final bacterial inoculum were plated on blood agar plates and incubated overnight at 37 °C to verify the amount of viable bacteria injected.

### Experimental procedures

Mice (*n* = 20–30 per time-point) were given a single intraperitoneal injection of 1 × 10^4^ CFU *E. coli* in 200 μl (range 1.0 × 10^4^–1.3 × 10^4^ CFU). Because this is a lethal dose and strain of *E. coli* (mice start dying around 20–22 h after injection) we treated all mice with ceftriaxone, an antimicrobial drug. Ceftriaxone (Fresenius Kabi, Den Bosch, the Netherlands) was intraperitoneally injected 12 and 24 h after *E. coli* injection in a dose of 20 mg/kg. In total 34 control mice (*n* = 6–8 per time-point) received 200 μl of pyrogen-free sterile isotonic saline via intraperitoneal injection; all control (non-infected) mice were treated with ceftriaxone. Inoculation was performed in three different rounds and mice were killed at 12 h, 2 days and 3 days after inoculation. An overview of experimental rounds, group size and time-points is illustrated in Additional file 1: Table S1.

Weight of all mice was monitored before inoculation, before administrating ceftriaxone/saline and before killing. Weight loss was considered a quantitative measurement for the degree of sickness. Additionally sickness behavior was monitored during experiments.

Mice were killed at the predetermined time-points. Blood and cerebral spinal fluid (CSF) were collected and spleen, median lobe of the liver and brain were harvested. Details of these proceedings are briefly explained in Additional file 1 and are described previously [11]. For the exact group sizes for the different analysis techniques, we refer to the figure legends in Results section and Additional file 1: Table S1.

### Isolating microglia for flow cytometry

Methods for isolating microglia are described in Additional file 1 and have been described previously [11]. Briefly, after overnight storage in Hibernate-A medium at 4 °C, left hemispheres of the brain were meshed through a cell strainer in a glucose–potassium–sodium buffer and were suspended in 1 ml enzyme buffer, followed by enzymatic digestion in collagenase type I. After enzymatic dissociation, cells were incubated in erythrocyte lysis buffer. Subsequently, cells were washed and resuspended in 20 ml Percoll of *ρ* = 1.03, then underlain with 10 ml Percoll of *ρ* = 1.095 and overlain with 5 ml of a glucose–potassium–sodium buffer. The tubes were centrifuged for 35 min at 1200×*g* at 20 °C, with slow acceleration and no break. The myelin layer on the top of the *ρ* = 1.03 phase was discarded and cells were collected from the interface between *ρ* = 1.095 and *ρ* = 1.03 Percoll. Next, cells were washed and counted with a Coulter counter. Approximately 1 × 10^5^ cells from every sample were collected and a portion of every sample was put in a pool tube, which subsequently was divided over 5 tubes for a blank sample and single stainings. Cells were stained in a total volume of 200 ul using antibodies with the following specificities: rat anti-mouse CD14 (immunoglobulin (Ig)G2a κ, clone Sa14-2, labeled with FITC, 1:800, BioLegend, Cat. 123301), rat anti-mouse CD11b (IgG2b κ, clone M1/70, labeled with PE, 1:200, BD Pharmingen, Cat. 557397), rat anti-mouse CD45 (IgG2b κ, clone 30-F11, labeled with APC, 1:500, eBioscience, Cat. 17-0451). To block nonspecific binding normal mouse serum (1:10) and anti-mouse CD16/CD32 (Alias Fcγ III/II receptor, IgG2b κ, clone 2.4G2, 1:100, BD Pharmingen, Cat. 553142) was added. About 10 min prior to fixation, 7-amino-actinomycin D (7-AAD) was added to each sample. After staining, cells were washed and fixated in 2% paraformaldehyde. Cells were washed and resuspended in 200 ul glucose–potassium–sodium buffer. Brains were perfused with PBS to prevent interference of circulating blood myeloid cells. In earlier experiments, we found no support for the presence of infiltrated myeloid cells (data shown in Additional file 1: Fig. S2). Therefore, all CD14-, CD11b- and CD45-positive cells were defined as microglial cells and were selected for flow cytometric analysis. Forward scatter (FCS) is proportional to the diameter of the cell, which can be used for discrimination of cells by size. Flow cytometric analysis was performed on a FACSCalibur machine (BD) and data were analyzed using FlowJo software version 7.6.1.

### Immunohistochemistry of murine brain

Histopathology was performed on the right brain hemisphere, fixed in 4% paraformaldehyde and paraffin-embedded. Coronal 5-μm-thick sections were cut and stained with hematoxylin and eosin (HE) according to standard procedures. Tissue sections were stained for ionized calcium-binding adaptor molecule 1 (Iba-1, rabbit polyclonal, 1:2000, Wako Pure Chemical Industries, Cat. 019-19741) using immunohistochemical procedures, as described previously [9]. Luxol fast blue–periodic acid–Schiff–hematoxylin staining was used to discriminate between white and gray matter and was compared to microglial staining to select different brain regions for analysis. In the end, slides were dehydrated and coverslipped with Pertex.

### Quantification of immunohistochemistry images

After staining all slides were scanned with a D. Sight fluo (A. Menarini, Florence, Italy) at 20× magnification. Two square millimeter of each digital image of cortex, hippocampus, thalamus and caudate nucleus was selected. Microglial cell bodies were manually counted twice by one observer (ICMH), who was blinded for all mice characteristics.

### Definition of microglial activation

Microglial cells were defined as activated based on the following criteria: (1) microglia showed an activated morphology based on immunohistochemical staining; (2) a significant increase in number, quantified with immunohistochemical staining; (3) a significant increase in expression of a microglial marker, quantified with flow cytometry (expression of CD45 and/or CD11b). When all three of the stated parameters were positive, microglial cells were defined as activated. When 1 or 2 parameters were positive, microglial cells were defined as moderately activated and when all parameters were negative, microglial cells were defined as inactive.

### RNA extraction and real time qPCR

The details of the analysis techniques are described in Additional file 1 and in our previous publication [11]. Data were analyzed using the Bio-Rad MyiQ Optical system Software version 1.0 and expression data were calculated using the deltaCt method. We investigated inflammatory response in brain homogenate by measuring messenger ribonucleic acid (mRNA) expression of general pro-inflammatory mediators: tumor necrosis factor alpha (TNF-α), interleukin 1 beta (IL-1β), interleukin 6 (IL-6), interleukin (IL-12) and high-mobility group 1 (HMGB1, which is a major mediator of inflammatory shock and is recognized as a damage-associated molecular pattern (DAMP) by certain immune cells, triggering inflammatory response); and immune regulatory mediators: monocyte chemotactic protein 1 (MCP-1) and macrophage colony-stimulating factor (M-CSF). Additionally, we examined myeloid cell markers: Iba-1 and CD11b. Lastly, we examined components of the Toll-like receptor (TLR) signaling cascade by measuring mRNA expression in brain homogenate of activators of the TLR signaling cascade; TLR-2 and mitogen-activated protein kinase 1 (MAPK-1); and inhibitors of the TLR signaling cascade; suppressor of cytokine signaling 1 (SOCS1) and deubiquitinase protein A20 (A20) [12]. In the systemic compartment, we investigated inflammatory response in spleen homogenate by measuring mRNA expression of TNF-α, IL-1β, HMGB1, IL-6, IL-12, M-CSF and MCP-1. Primer sequences are depicted in Additional file 1: Table S2.

### Statistical analysis

A parametric or non-parametric two-way ANOVA (factors: genetics and time-point) was conducted on all data (flow cytometry, immunohistochemistry and mRNA expression data), depending on sample size and distribution of the data. For non-parametric ANOVAs, ranked transformation of data was performed. Subsequently continuous variables were tested with post hoc Mann–Whitney *U* tests or Student’s *t*-tests. The assumptions of normality and homoscedasticity were tested with the Shapiro–Wilk and Levene’s test, respectively. Statistical significance was set to *p* ≤ 0.05. Because of the exploratory nature of this study, correction for multiple comparisons was not applied. All statistic test were done using IBM SPSS Statistics (version 26.0). For visual presentation of quantitative results, GraphPad Prism was used (GraphPad Software, version 6.07, La Jolla, CA, USA).

## Results

### Mouse models

At 12 h after inoculation all infected mice showed signs of sickness (Additional file 1: Fig. S3). Ceftriaxone treatment was administrated at 12 h and 24 h after inoculation. Behavior turned to normal at 30 h after inoculation in all infected mice, with the exception of 1 infected WT and 1 infected *α7nAChR*^*−/−*^ mice—these mice died before the predefined time-point. Infected *α7nAChR*^*−/−*^ mice lost more weight compared to WT mice at 12 h and 3 days after inoculation. Blood, spleen and liver cultures were positive at 12 h, 2 days and 3 days after inoculation in both WT as *α7nAChR*^*−/−*^ mice, which means the systemic infection was not completely cleared in both groups and systemic infection was ongoing. CSF cultures were negative for both groups at all time-points, which shows mice did not develop secondary meningitis (Additional file 1: Fig. S4).

### Microglial activation

**Fig. 1:**
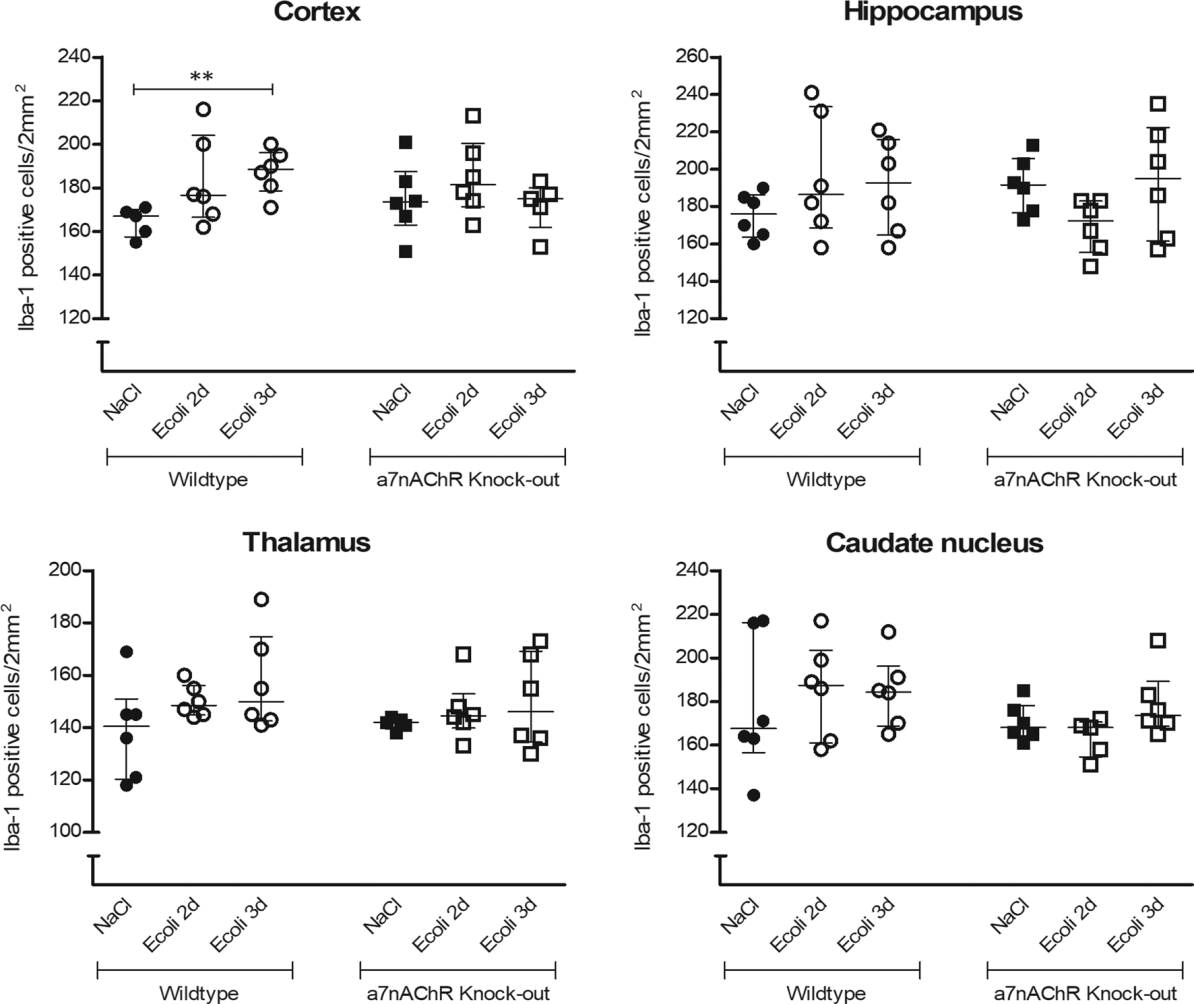
Histopathological counts of microglia. Number of Iba-1 positive microglial cell bodies per experimental group (Control WT: *n* = 5–6; Infected WT: *n* = 5–6; Control *α7nAChR*^*−/−*^: *n* = 5–6; Infected *α7nAChR*^*−/−*^: *n* = 5–6) in different brain regions (cortex, hippocampus, thalamus and caudate nucleus) per 2 mm^2^. Non-parametric two-way ANOVA (factors: age and time-point) was conducted for all brain regions and continuous variables were tested with post hoc Mann–Whitney *U* tests. Ranked transformation of data was performed. Data represent median ± IQR, **p ≤ 0.01

**Fig. 2:**
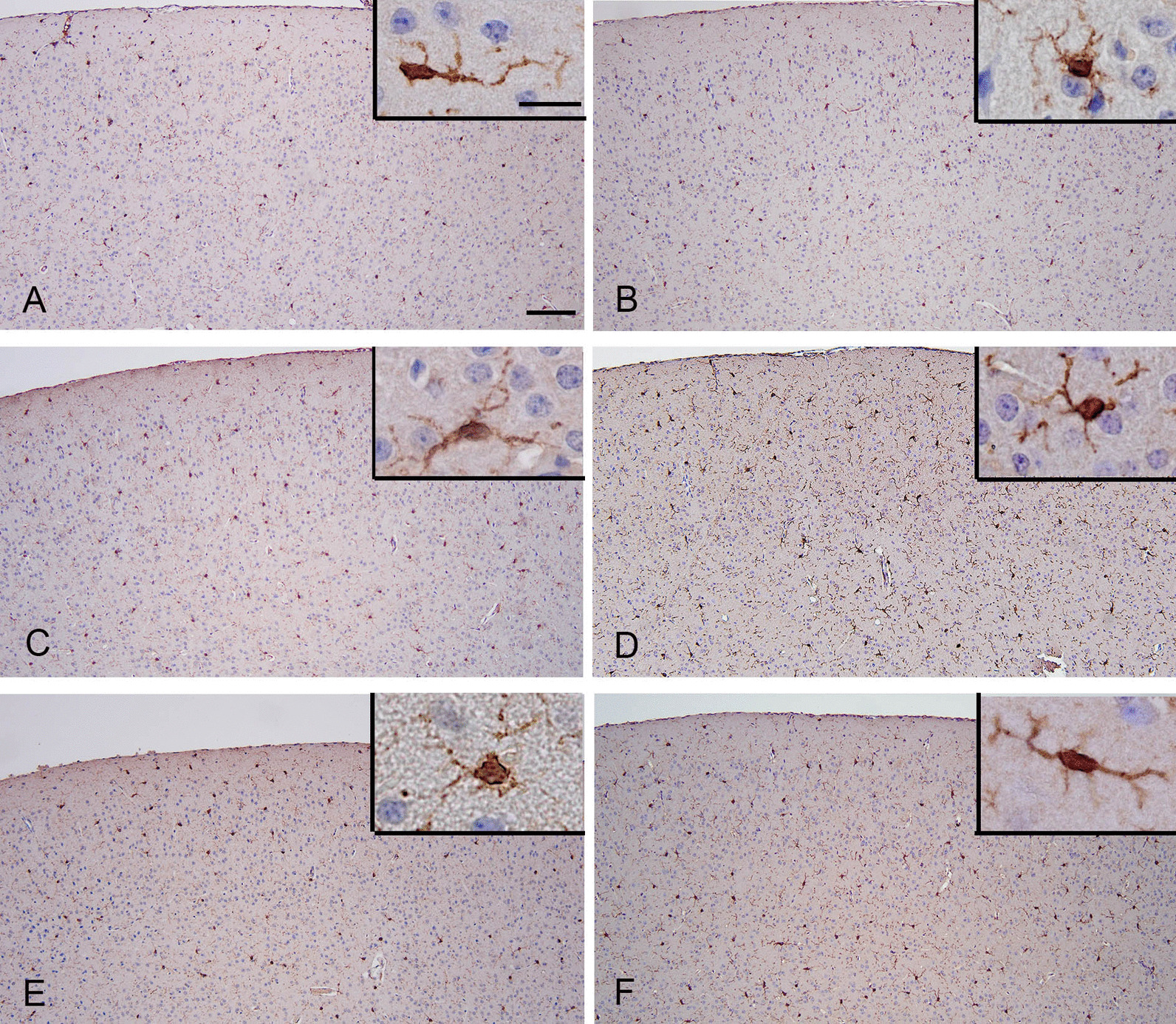
Representative images of immunohistochemical staining with Iba-1 antibody of cortex region for control WT mice (**A**), control *α7nAChR*^*−/−*^ mice (**B**), infected WT mice, killed at day 2 (**C**), infected *α7nAChR*^*−/−*^ mice, killed at day 2 (**D**), infected WT mice, killed at day 3 (**E**), and infected *α7nAChR*^*−/−*^ mice, killed at day 3 (**F**). Scale bar 100 micronM, insert 20 micronM

**Fig. 3:**
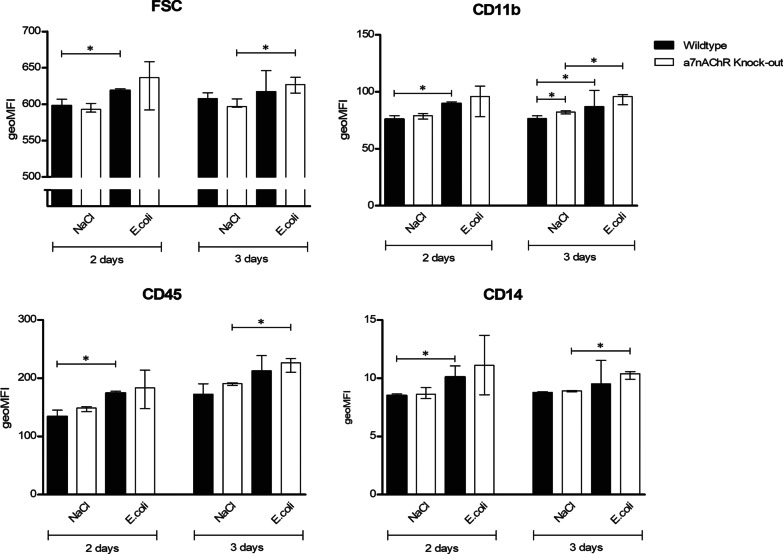
Geometric means (geoMFI) for forward scatter (FSC), expression of cluster of differentiation (CD) 11b and CD45 and CD14 measured with flow cytometry. Mann–Whitney *U* tests were performed. Data represent median ± IQR, *p ≤ 0.05. NB: Flow cytometry for every time-point was done on a different day. Laser characteristics vary per day, and therefore the geoMFIs are not comparable between the different time-points. As such, every infected group (*n* = 3–4) has its own control group (*n* = 4–5)

In infected WT mice, immunohistochemistry of Iba-1 positive cells showed an increase in cell number in cortex compared to uninfected WT mice at day 3 after inoculation (median 189 cells/2 mm^2^ (interquartile range [IQR] 179–196) vs 167 cells/2 mm^2^ (IQR 158–170); *p* = 0.002) without morphological changes of the cells. No increase in cell number and no morphological changes were seen in hippocampus, thalamus and caudate nucleus in infected WT mice compared to uninfected WT mice at day 2 and day 3 after inoculation (Figs. 1 and 2). Flow cytometry of isolated microglial cells of infected WT mice at day 2 after inoculation showed an increase in forward scatter (FSC) (*p* = 0.03), increased expression of CD11b (*p* = 0.04), CD45 (*p* = 0.04) and CD14 (*p* = 0.04) compared to uninfected WT mice. At day 3 after inoculation there was an increase in expression of CD11b (*p* = 0.03) in infected WT mice compared to uninfected WT mice (Fig. 3). In infected *α7nAChR*^*−/−*^ mice, immunohistochemistry of Iba-1-positive cells showed no increase and no morphological changes in cortex, hippocampus, thalamus and caudate nucleus as compared to WT mice (Figs. 1 and 2). Flow cytometry of isolated microglial cells of infected *α7nAChR*^*−/−*^ mice at day 3 after inoculation showed an increase in forward scatter (*p* = 0.04), increased expression of CD11b (*p* = 0.02), CD45 (*p* = 0.02) and CD14 (*p* = 0.02) compared to uninfected *α7nAChR*^*−/−*^ mice (Fig. 3).

Comparing uninfected and infected WT and *α7nAChR*^*−/−*^ mice, immunohistochemistry showed no differences in Iba-1 positive cell number or morphology in the four different brain regions (Figs. 1 and 2). There was no interaction effect for genetic background and time-point, indicating no different course of Iba-1-positive cell number in time for the different genetic backgrounds in the different brain regions. Flow cytometry showed an increased expression of CD11b in uninfected *α7nAChR*^*−/−*^ mice compared to uninfected WT mice (*p* = 0.03). No other differences were seen in forward scatter or expression of CD11b, CD45 and CD14 comparing WT and *α7nAChR*^*−/−*^ mice (Fig. 3). Furthermore, the tendency for all flow cytometry parameters in WT and *α7nAChR*^*−/−*^ mice after infection was the similar and no interaction effects were seen.

### Inflammatory mediators in brain

Our main focus was to compare inflammatory mediators in brain of WT mice and *α7nAChR*^*−/−*^ mice. Therefore, we only describe the differences between WT mice and *α7nAChR*^*−/−*^ mice here, for a full report and figures of all measured inflammatory mediators in brain, we refer to Additional file 1: Results, Figures S5–S8.

**Fig. 4:**
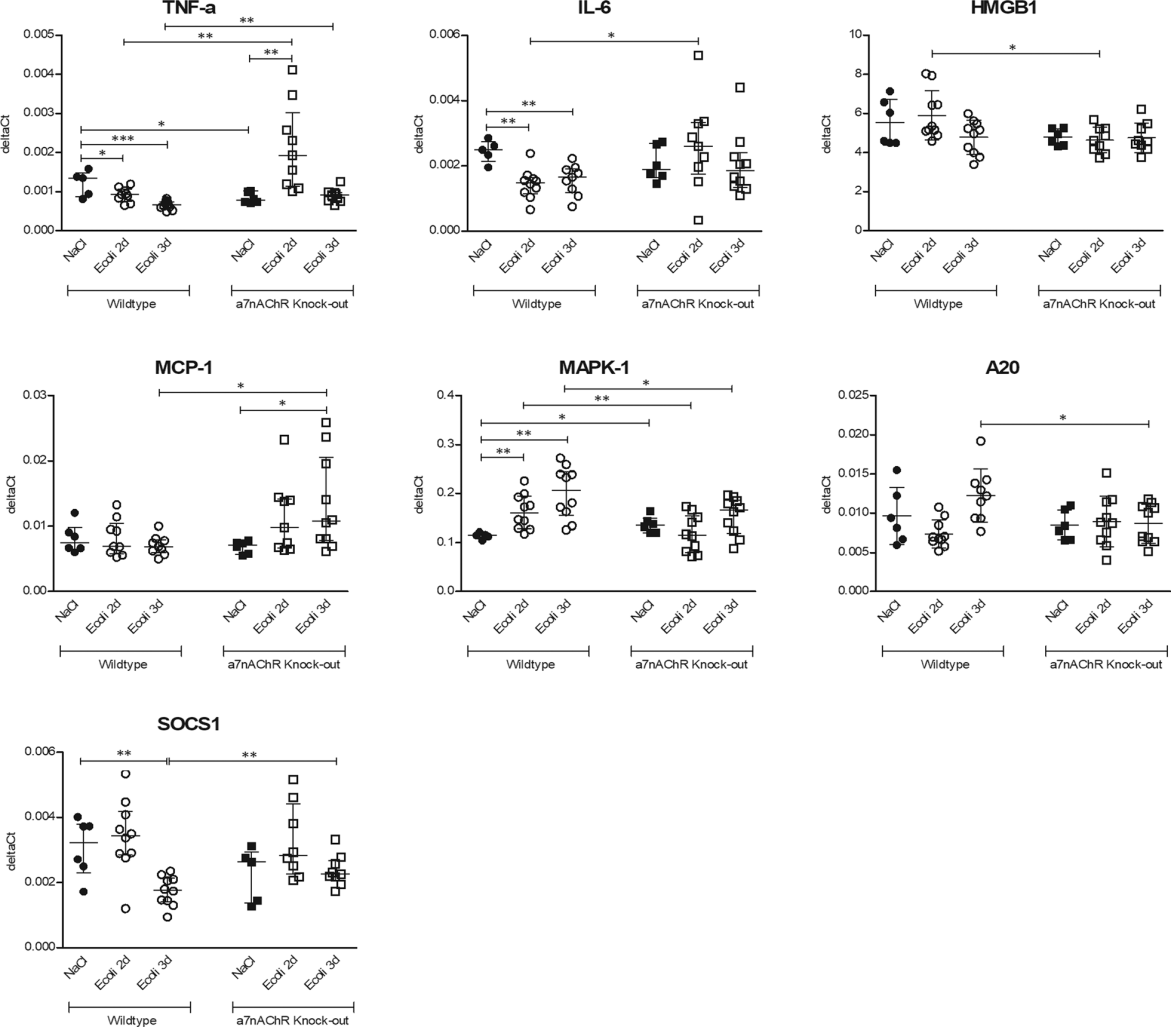
Expression of brain homogenate of inflammatory mediators TNF-α, IL-6, HMGB1, MCP-1, MAPK-1, A20 and SOCS1 mRNA’s, illustrated in deltaCts. Group size of control groups varies from *n* = 5 to 6, groups size of infected groups varies from *n* = 9 to 10. Parametric two-way ANOVA (factors: genetics and time-point) were conducted and continuous variables were tested with post hoc Student’s *t*-tests for HMGB1 and A20. Data represent mean ± SD. Non-parametric two-way ANOVA was conducted and continuous variables were tested with post hoc Mann–Whitney *U* tests for TNF-α, IL-6, MCP-1, MAPK-1 and SOCS1. For non-parametric ANOVAs, the ranked transformation of data was performed. Data represent median ± IQR. *p ≤ 0.05, **p ≤ 0.01, ***p ≤ 0.001

Analysis of brain mRNA expression levels (Fig. 4) in uninfected WT mice showed increased expression of TNF-α and MAPK1 mRNA’s compared to uninfected *α7nAChR*^*−/−*^ mice (resp. *p* = 0.03; *p* = 0.02). At 2 days after inoculation infected WT mice showed increased expression of HMGB1 and MAPK1 mRNA’s (resp. *p* = 0.03; 0.01) and decreased expression of TNF-α and IL-6 mRNA’s (resp. *p* = 0.005; *p* = 0.02) compared to infected *α7nAChR*^*−/−*^ mice. At 3 days after inoculation infected WT mice showed increased expression of MAPK1 and A20 mRNA’s (resp. *p* = 0.03; *p* = 0.02) and decreased expression of TNF-α, MCP-1 and SOCS1 mRNA’s (resp. *p* = 0.002; *p* = 0.02; *p* = 0.01) compared to infected *α7nAChR*^*−/−*^ mice. For TNF-α and IL-6 there was an interaction effect for genetic background an time-point (resp. *p* < 0.0001; *p* = 0.02). This indicates a different course of expression of TNF-α and IL-6 mRNA’s in time for WT and *α7nAChR*^*−/−*^ mice, where in WT mice there was a decrease for both mediators at day 2 and day 3 after inoculation, while in *α7nAChR*^*−/−*^ mice there was an increase for both mediators at day 2 after inoculation. There was an interaction effect for expression of MAPK1 and A20 mRNAs (resp. *p* = 0.01; *p* = 0.02), where in infected WT mice there was an increased expression in time compared to uninfected WT mice, while in infected *α7nAChR*^*−/−*^ mice there was no change in expression of MAPK1 or A20 mRNA’s compared to uninfected *α7nAChR*^*−/−*^ mice.

### Inflammatory mediators in spleen

**Fig. 5:**
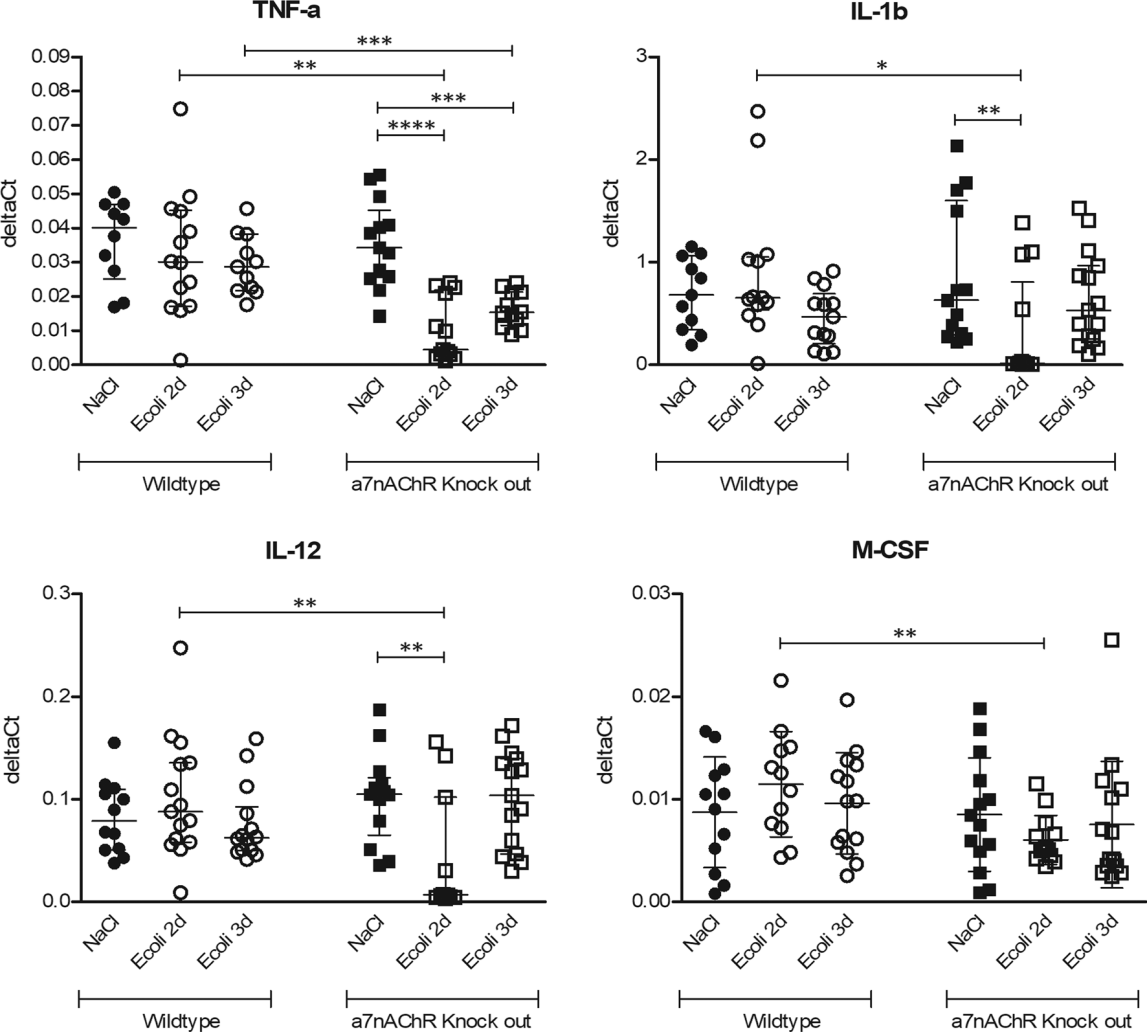
Expression of spleen homogenate of general pro-inflammatory mediators TNF-α, IL-1β, IL-12 and M-CSF mRNA’s, illustrated in deltaCts. Group size of control groups varies from *n* = 10 to 14, groups size of infected groups varies from *n* = 11 to 15. Parametric two-way ANOVA (factors: genetics and time-point) was conducted and continuous variables were tested with post hoc Student’s *t*-tests for M-CSF. Data represent mean ± SD. Non-parametric two-way ANOVA was conducted and continuous variables were tested with post hoc Mann–Whitney *U* tests for TNF-α, IL-1β, IL-12. For non-parametric ANOVAs, the ranked transformation of data was performed. Data represent median ± IQR. *p ≤ 0.05, **p ≤ 0.01, ***p ≤ 0.001, ****p ≤ 0.0001

Comparing inflammatory mediators in spleen (Fig. 5) between infected WT and *α7nAChR*^*−/−*^ mice, WT mice showed increased expression of TNF-α, IL1-β, IL-12 and M-CSF mRNA’s at day 2 after inoculation (resp. *p* = 0.002; *p* = 0.02; *p* = 0.01; *p* = 0.007). At day 3 after inoculation there was increased expression of TNF-α mRNA in infected WT mice (*p* = 0.0006) compared to infected *α7nAChR*^*−/−*^ mice. There was an interaction effect for genetic background and time-point for expression of TNF-α, IL-1β, IL-6 and IL-12 mRNA’s (resp. *p* = 0.009, *p* = 0.01; *p* = 0.02; *p* = 0.004). For all parameters there was a decreased expression at day 2 in infected *α7nAChR*^*−/−*^ mice compared to uninfected *α7nAChR*^*−/−*^ mice, while in infected WT mice there was no change in mRNA expression for these parameters compared to uninfected WT mice.

## Discussion

Our results show that loss of function of α7nAChR during systemic infection led to an increased expression of TNF-α and IL-6 in brain after systemic infection with *E. coli*, but not to differences in microglial cell number or morphological activation of microglia. Microglial cells are not the only resident immune cells in the CNS expressing α7nAChRs. The same receptor is also expressed on neurons in cortex and hippocampus and on non-neuronal cells, including microglial cells, astrocytes, oligodendrocytes and brain endothelial cells [13], whereas astrocytes, next to microglial cells, are known to be important players in cerebral innate immunity [14]. Following activation, astrocytes can secrete inflammatory mediators, such as TNF-α, IL-6 and MCP-1, the latter also known as chemokine (C–C motif) ligand 2 (CCL2) [14]. Astrocytes could be responsible for the increase in mRNA expression of TNF-α, IL-6, MCP-1 we found in brain homogenate in infected *α7nAChR*^*−/−*^ mice as compared to infected WT mice.

The finding of lower expression in brain for HMGB-1 and MAPK1 mRNA’s and lower expression in spleen for TNF-α and IL-1β mRNA’s in infected *α7nAChR*^*−/−*^ mice compared to infected WT mice was unexpected. Several studies showed that cholinergic agonists inhibit HMGB1 and MAPK1 release [15–18] and when human macrophages are exposed to nicotine or acetylcholine, production of systemic TNF-α and IL-1β after stimulation with endotoxin is inhibited [4]. Furthermore, during septic peritonitis, initial pro-inflammatory cytokine release in peritoneal lavage fluid, was enhanced after previous vagotomy and was decreased after nicotine pretreatment, independently of the integrity of the vagal nerve [19]. However, the aim of these studies was to evaluate the potential of cholinergic stimulation as a possible therapy for severe sepsis, and none of these studies used knock-out mice or removed the α7nAChR in another way. To study the role of the endogenous cholinergic anti-inflammatory pathway in antibacterial defense, rather than the effect of exogenous stimulation, Giebelen et al. induced an *E. coli* peritonitis in *α7nAChR*^*−/−*^ mice [20]. This model was similar to our study, except they chose to withhold antibiotic treatment. They showed that *α7* deficiency was associated with an accelerated bacterial clearance of *E. coli* at 20 h after infection in peritoneal lavage fluid, blood, liver, spleen, kidney and lung, preceded by a more robust recruitment of neutrophils to the primary site of infection. Furthermore, they showed lower plasma levels of pro-inflammatory mediators, such as TNF-α, IL-6 and IL-12, in infected *α7*-deficient mice at 20 h compared to infected WT mice. These results confirm that *α7* deficiency results in a different interpretation of the role of the α7nAChR in inflammatory response, than the role of this receptor after exogenous stimulation during inflammation. Another study investigated the role of α7nAChRs on microglia in vitro, using short hairpin RNA (shRNA) knock-down expression of α7nAChRs cultured microglial cells [21]. After stimulation with LPS, the changes in IL-1β, IL-6, IL-4 and IL-10 production were similar for knock-down and control microglial cells [21]. However, after stimulation with acetylcholine, LPS-induced IL-1β and IL-6 secretion was inhibited but acetylcholine failed to reverse the increase of LPS-induced IL-1β and IL-6 in the knock-down microglial cells. This could indicate that cells which lack α7nAChRs initially react the same to an inflammatory response as controls, because of the possible development of other regulatory mechanisms. For example, it has been shown that α9 and α10 subunits are upregulated in brain of *α7nAChR*^*−/−*^ mice and both α7 and α9 are down-regulated in brain of *α10nAChR*^*−/−*^ mice [22]. Furthermore, in retina of *α7nAChR*^*−/−*^ mice the absence of the α7nAChR was associated with complex layer-specific changes in the expression of AChR subunits and subtypes [23]. This proves that adaptive neurobiological changes occur during development of a knock-out animal, that result in compensatory mechanisms in response to the deletion of α7nAChRs.

Our study has several limitations. First, the age of the mice used in these experiments; we hypothesized an increase in microglial cell number and transformation of microglial cells to an activated morphology in infected *α7nAChR*^*−/−*^ mice. Our results do not exclude a role of the cholinergic anti-inflammatory pathway in the neuro-inflammatory pathway after systemic infection. In normal ageing, microglial cells exhibit a “primed” phenotype, were they show an enhanced inflammatory response to an inflammatory stimulus [24]. Primed microglial cells show upregulation of genes associated with antigen presentation [25, 26], an increase in pro-inflammatory cytokines [27] and suppression of anti-inflammatory genes [26]. Cholinergic regulation of glial cells via α7nAChRs receptors control the secretion of inflammatory mediators and normal ageing is associated with the reduction of these receptors in de CNS [28]. While the reduction of α7nAChRs during ageing is most likely associated with microglial priming and is a potential contributor to impaired regulatory signaling, ageing is a complex physiological process and microglial cells have numerous regulatory mediators, such as fractalkine (CX3CL1), triggering receptor expressed on myeloid cells 2 (TREM2) and CD200 [26, 29, 30], which might be impaired during ageing. The mice in our experiments were young and therefore had no primed phenotype of microglial cells, therefore the role of the anti-cholinergic anti-inflammatory pathway might be bigger with advancing age where microglial cells are primed and other regulatory pathways may be impaired. Second, the chosen time-points; our focus is on protracted neuro-inflammation after systemic infection, therefore including a later time-point then 3 days could be informative. Third, one could argue the fact of using solely male mice, while evidence indicates that the number and phenotype of microglial cells differ between sex [31]. However, because of the exploratory nature of this study, we wanted to avoid the influence of the hormonal cycle of female mice, in order to limit variations not related to inflammatory processes.

## Conclusion

In conclusion, loss of function of α7nAChR during systemic infection did not influence microglial cell number or morphological activation of microglia. We did observe increased expression of TNF-α and IL-6 in brain after systemic infection with *E. coli* that could be explained by activation of other cell types than microglia.

## Supplementary Information

**Additional file 1.** Appendix.

## Data Availability

The datasets used and/or analyzed during the current study are available from the corresponding author on reasonable request.

## Abbreviations

α7nAChR: α7 Subunit of the nicotinic ACh receptor
*α7nAChR*^*−*^^*/*^^*−*^: α7NAChR knock out
A20: Deubiquitinase protein A20
Ach: Acetylcholine
CCL2: C–C motif ligand 2
CFU’s: Colony-forming units
CX3CL1: Fractalkine
deltaCt: Delta cycle threshold
HE: Hematoxylin and eosin
HMGB1: High-mobility group 1
IQR: Interquartile range
Iba-1: Ionized calcium-binding adaptor molecule 1
LPS: Lipopolysaccharide
LB: Luria–Bertani medium
MAPK1: Mitogen-activated protein kinase
M-CSF: Macrophage colony-stimulating factor
MCP-1: Monocyte chemotactic protein 1
SOCS1: Suppressor of cytokine signaling 1
TLR: Toll-like receptor
TNF-α: Tumor necrosis factor alpha
TREM2: Triggering receptor expressed on myeloid cells 2
WT: Wild-type
