# Supplementary material for: Microglial cell response in α7 nicotinic acetylcholine receptor-deficient mice after systemic infection with Escherichia coli
Source: J Neuroinflammation. 2022 Apr 12;19:94. doi: 10.1186/s12974-022-02452-8 (PMC9006549; doi:10.1186/s12974-022-02452-8)
Supplement: Supplementary file 1 — Additional file 1. Appendix. [file 12974_2022_2452_MOESM1_ESM.docx]

**APPENDIX**

**METHODS**

*Sacrificing the mice*

Mice were anesthetized by intraperitoneal injection of ketamine (190 mg/kg, Eurovet Animal Health, Bladel, the Netherlands) and medetomidine (0.3 mg/kg, Pfizer Animal Health, Capelle aan den IJssel, the Netherlands). Body weight was assessed, after which cardiac puncture for blood collection followed. Blood was collected in sterile tubes containing EDTA and stored on ice. The abdomen was opened and the vena cava was severed, subsequently cerebral spinal fluid (CSF) was collected by puncture of the cisterna magna, collected in sterile tubes and stored on ice. Thereafter the thorax was opened and perfusion of organs with sterile phosphate buffered saline (PBS) was performed via the left cardiac ventricle (approximately 20 ml PBS in 5 minutes). The spleen and median lobe of the liver were taken up in 20% weight per volume sterile saline. The right hemisphere of the brain was either suspended in 10% buffered formalin and embedded in paraffin for histopathology or taken up in 20% weight per volume sterile saline. The left hemisphere of the brain was either suspended in 5 ml of Hibernate-A medium (Invitrogen, Cat. A12475-01) and stored by 4 degrees Celsius (°C) (for isolating microglial cells for flowcytometry) or taken up in 20% weight per volume sterile saline (for RNA extraction and real time PCR). The organs suspended in sterile saline were put on ice and were disrupted with a tissue homogenizer. Directly after homogenizing the organs, 50 μl of the tissue homogenate was suspended in 350 μl RA1 lysis buffer (Kit content of NucleoSpin® RNAII, Macherey-Nagel, Cat. 740955) and stored at – 80°C for messenger ribonucleic acid (mRNA) isolation. CSF was diluted 1:100 in sterile saline because of the low volumes. Serial ten-fold dilutions of blood, CSF, liver and spleen homogenates were plated on blood agar plates and bacteria were allowed to grow overnight at 37°C.

|  |  | Number of mice per experimental group | |
| --- | --- | --- | --- |
| Round | Time point | NaCl+Ceft (Wildtype/a7nAChR KO) | *E. coli*+Ceft (Wildtype/ a7nAChR KO) |
| 1 | t=12hours | 6 (3/3) | 20 (10/10) |
| 2 | t=2days | 8 (4/4) | 10 (5/5) |
|  | t=3days | 8 (4/4) | 10 (5/5) |
| 3 | t=2days | 6 (3/3) | 20 (10/10) |
|  | t=3days | 6 (3/3) | 20 (10/10) |

Table S1: Group size per experiment round and time point. Ceft=Ceftriaxone, a7nACh=a7 nicotinic acetylcholine receptor, KO=Knock-out

*Isolating microglia for flow cytometry*

The overnight storage of brain tissue for flow cytometry was necessary because we lacked the manpower to process all the tissue on the same day as we sacrificed the animals. The quality of microglial cells was tested in other experiments and the overnight storage had no influence on the results (data shown in Appendix, Figure S1). After overnight storage in Hybernate-A medium at 4°C, left hemispheres (approximately weighing 250 mg) were meshed through a 70 μm cell strainer (nylon, BD Falcon, Cat. 352350) in a glucose-potassium-sodium buffer (8 g/l NaCl, 0.4 g/l KCl, 1.77 g/l Na_2_HPO_4_.2H_2_O, 0.69 g/l NaH_2_PO_4_.H_2_O, 2 g/l D-(1)-glucose, pH 7.4) with 0.3% bovine serum albumin (Roche, Cat. 10735108001) and collected in 50 ml tubes. After centrifuging (1400 rpm, 7 minutes, 4°C), cell pellets were suspended in 1 ml enzyme buffer (4 g/l MgCl_2_, 2.55 g/L CaCl_2_, 3.73 g/L KCl, and 8.95 g/L NaCl, pH 6–7), followed by enzymatic digestion in collagenase type I (370 units, Worthington, Cat. 9001-12-1) and DNase I (10 mg/ml, Roche, Cat. 1284932) for 45 minutes, at 37°C, while shaking. After enzymatic dissociation, cells were washed with glucose-potassium-sodium buffer and incubated for 2 minutes on ice in 2 ml cold erythrocyte lysis buffer (8.3 g/l NH_4_Cl, 1 g/l KHCO_3_ and 0.03 g/l EDTA, pH 7.4). Subsequently, cells were washed and resuspended in 20 ml Percoll (GE healthcare, Cat. 17-0891-01) of ρ=1.03, then underlain with 10 ml Percoll of ρ=1.095 and overlain with 5 ml of glucose-potassium-sodium buffer. The tubes were centrifuged for 35 minutes at 1200xg at 20°C, with slow acceleration and no break. The myelin layer on the top of the ρ=1.03 phase was discarded and cells were collected from the interface between ρ=1.095 and ρ=1.03 Percoll. Next, cells were washed and counted with a Coulter counter (Beckman Coulter, Z2). Approximately 1x10^5^ cells from every sample were transferred into separate polystyrene coated round bottomed 5 ml tubes (BD Falcon, Cat. 352008). Depending on cell numbers, a portion of every sample was put in a pool tube, which subsequently was divided over 5 tubes for a blanc sample and single stainings, every tube contained approximately 1x10^5^ cells.

Cells were stained in a total volume of 200 ul using antibodies with the following specificities: rat anti-mouse CD14 (immunoglobulin (Ig)G2a κ, clone Sa14-2, labeled with FITC, 1:800, BioLegend, Cat. 123301), rat anti-mouse CD11b (IgG2b κ, clone M1/70, labeled with PE, 1:200, BD Pharmigen, Cat. 557397), rat anti-mouse CD45 (IgG2b κ, clone 30-F11, labeled with APC, 1:500, eBioscience, Cat. 17-0451). To block aspecific binding normal mouse serum (1:10) and anti-mouse CD16/CD32 (Alias Fcγ III/II receptor, IgG2b κ, clone 2.4G2, 1:100, BD Pharmigen, Cat. 553142) was added. Cells were incubated with antibodies for 30 minutes on ice in polystyrene coated round bottomed 5 ml tubes. About 10 minutes prior to fixation, 2.5 ul of 7-amino-actinomycin D (7-AAD, labeled with PerCP, 1:80 concentration, BD Pharmigen, Cat. 559925) was added per sample. After staining, cells were washed and fixated in 2% paraformaldehyde for 10 minutes on ice. Cells were washed and resuspended in 200 ul glucose-potassium-sodium buffer. As stated above, all mice brain were perfused with PBS to prevent interference of circulating blood myeloid cells. In earlier experiments we found no support for the presence of infiltrated myeloid cells (data shown in Appendix Figure S2). Therefore, all CD14-, CD11b- and CD45-positive cells could be defined as microglial cells and were selected for flow cytometric analysis. Flow cytometric analysis was performed on a FACSCalibur machine (BD) and data were analyzed using FlowJo software version 7.6.1.


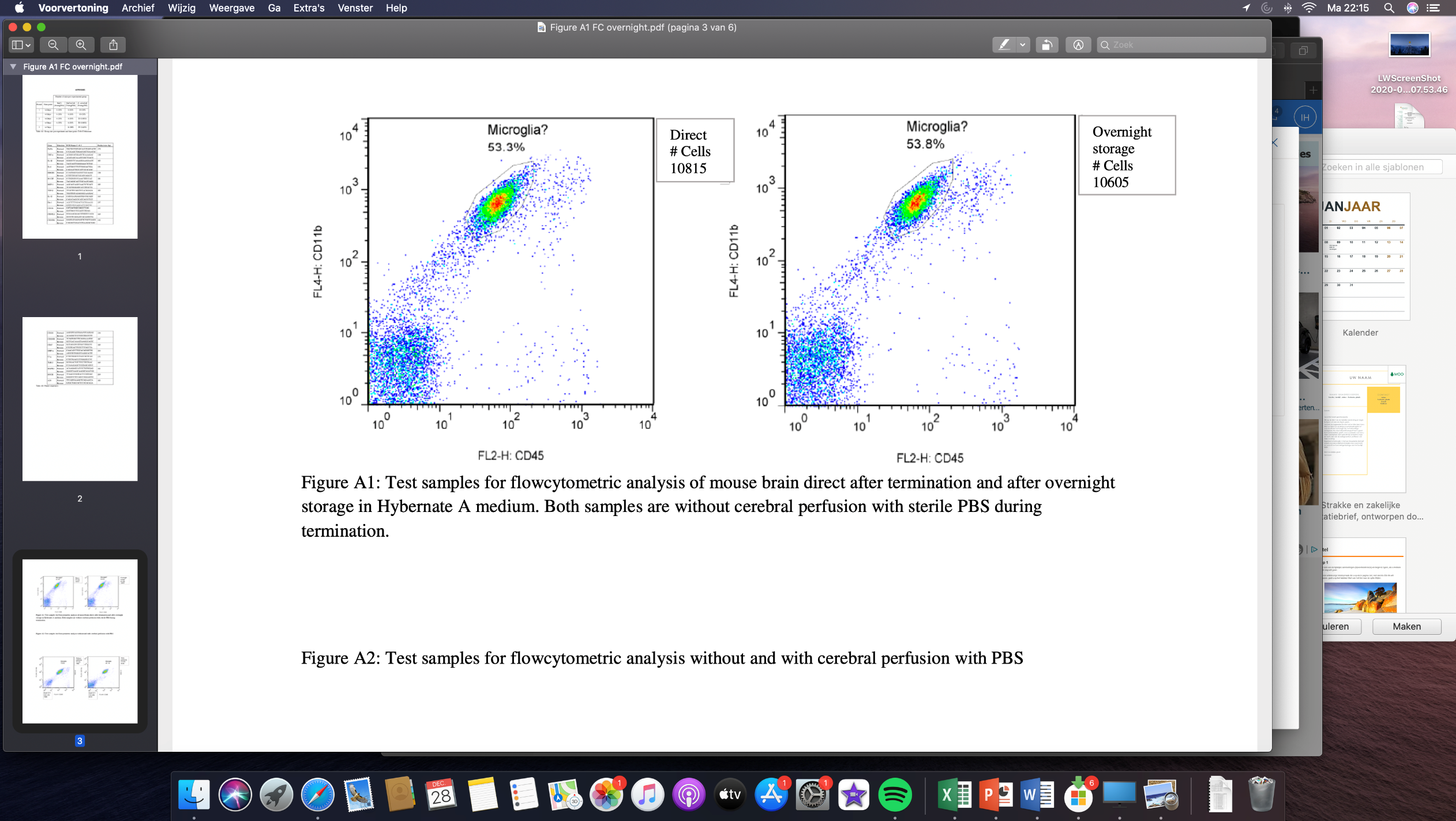


Figure S1: Test samples for flowcytometric analysis of mouse brain direct after termination and after overnight storage in Hybernate A medium. Both samples are without cerebral perfusion with sterile PBS during termination.

**
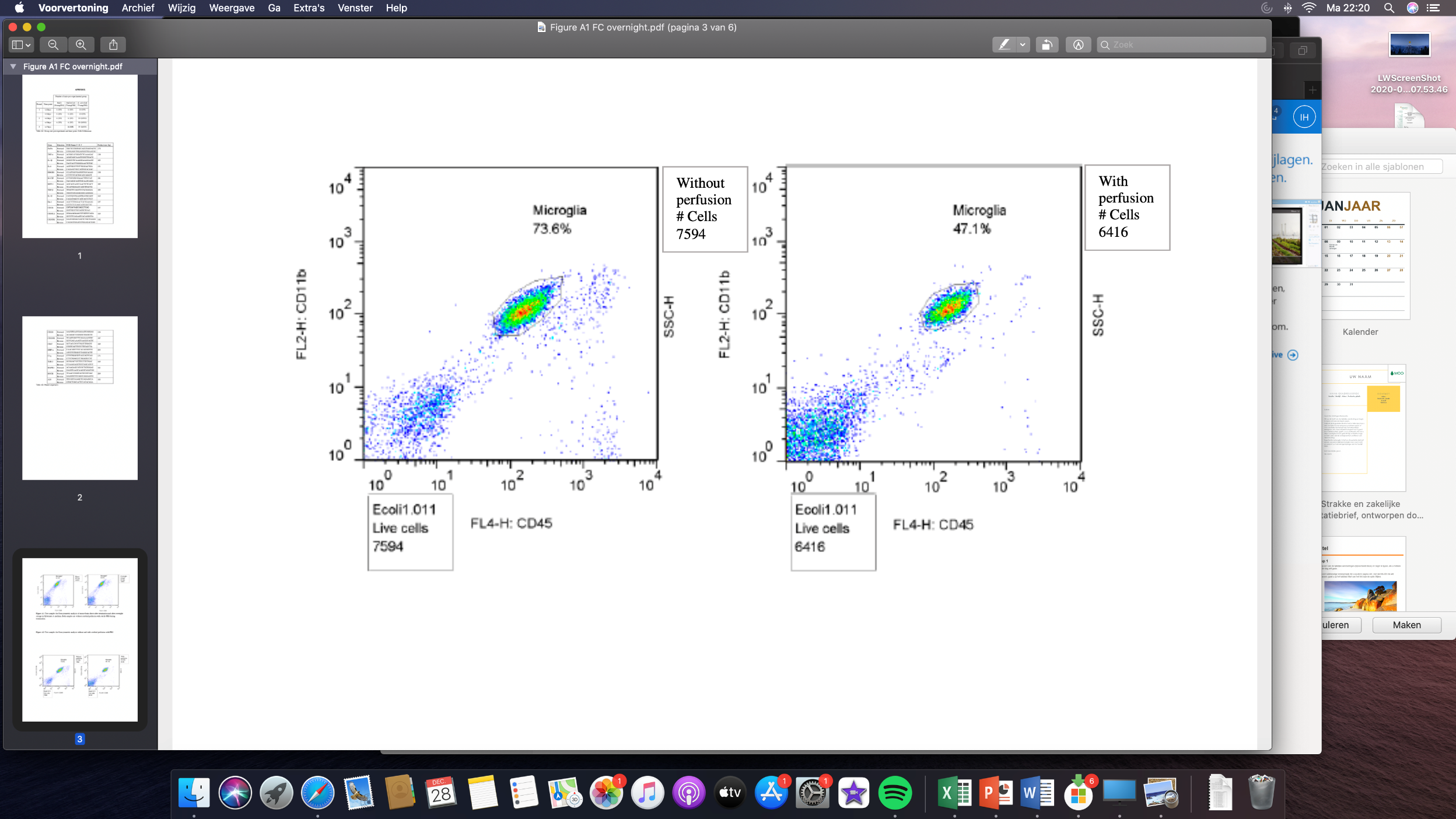
**

Figure S2: Test samples for flowcytometric analysis without and with cerebral perfusion with PBS

*RNA extraction and real time qPCR*

Total RNA was extracted from murine brain and spleen homogenates using the nucleospin II extraction kit (Macharey-Nagel GmbH, Duren, Germany). The concentration of the RNA was measured using Nanodrop Spectrophotometer (Nanodrop Technologies, USA) and the purity was assessed by the ratio of absorbance at 260 and 280 nm. RNA purity was within range of 2.0-2.1. Complementary deoxyribonucleic acid (cDNA) was synthesized from equal amounts of RNA using Iscript^tm^ according to the manufacturer’s protocol (Biorad Laboratories, Hercules, USA). Gene-specific analysis by real-time quantitative polymerase chain reaction (qPCR) was performed using an iCycler MyiQTM system with Bio-Rad iQ^tm^SYBRGreenSupermix (Biorad Laboratories, Hercules, USA). Expression levels were normalized to reference gene Non-POU-domain containing octamer binding protein (NoNo). Primer sequences are depicted in appendix table S2. A negative control without the Reverse Transcriptase was also used. Data were analyzed using the Bio-Rad MyiQ Optical system Software version 1.0 and expression data were calculated using the deltaCt method.

| Gene | Direction | PCR Primer 5' 🡪 3' | Product size (bp) |
| --- | --- | --- | --- |
| NoNo | Forward | TGCTCCTGTGCCACCTGGTACTC | 170 |
|  | Reverse | CCGGAGCTGGACGGTTGAATGC |  |
| TNF-α | Forward | ACGGCATGGATCTCAAAGAC | 138 |
|  | Reverse | AGATAGCAAATCGGCTGACG |  |
| IL-1β | Forward | GGGCCTCAAAGGAAAGAATC | 183 |
|  | Reverse | TACCAGTTGGGGAACTCTGC |  |
| IL-6 | Forward | AGTTGCCTTCTTGGGACTGA | 191 |
|  | Reverse | CAGAATTGCCATTGCACAAC |  |
| HMGB1 | Forward | CCATTGGTGATGTTGCAAAG | 158 |
|  | Reverse | CTTTTTCGCTGCATCAGGTT |  |
| M-CSF | Forward | CCTGTGTCCGAACTTTCCAT | 181 |
|  | Reverse | TACAGGCAGTTGCAATCAGG |  |
| MCP-1 | Forward | AGCACCAGCCAACTCTCACT | 185 |
|  | Reverse | TCATTGGGATCATCTTGCTG |  |
| TGF-β | Forward | TTGCTTCAGCTCCACAGAGA | 183 |
|  | Reverse | TGGTTGTAGAGGGCAAGGAC |  |
| IL-12 | Forward | CATCGATGAGCTGATGCAGT | 163 |
|  | Reverse | CAGATAGCCCATCACCCTGT |  |
| Iba-1 | Forward | AGCTTTTGGACTGCTGAAGG | 197 |
|  | Reverse | GTTTCTCCAGCATTCGCTTC |  |
| CD11b | Forward | CATCAATAGCCAGCCTCAG | 197 |
|  | Reverse | GGTTGCCTCCAGTCTCAG |  |
| TLR-2 | Forward | GCGGACTGTTTCCTTCTGAC | 165 |
|  | Reverse | CCAAAGAGCTCGTAGCATCC |  |
| MAPK1 | Forward | ACAAGAGCATCCCTGTGGAG | 161 |
|  | Reverse | GAGGTAAGCAAGGCAGATGG |  |
| SOCS1 | Forward | TTAACCCGGTACTCCGTGAC | 209 |
|  | Reverse | GAGGTCTCCAGCCAGAAGTG |  |
| A20 | Forward | TTCATCGAAGCTCAGAACCA | 183 |
|  | Reverse | GTGCTGGCACTCCATACAGA |  |

Table S2: Primer sequences.

**RESULTS**


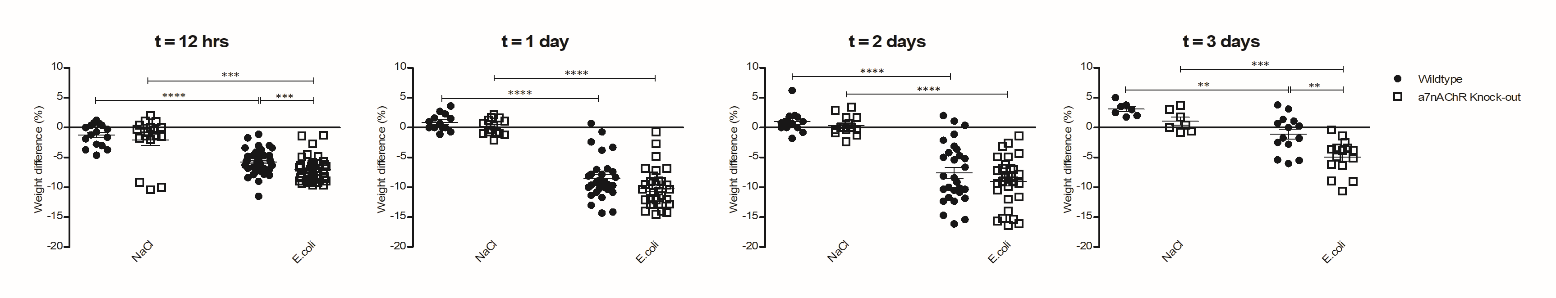


Figure S3: Weight difference per time point in percentage. Student t-tests were performed, data represent mean + SD, *P<0.05, **P<0.01, ***P<0.001, ****P<0.0001


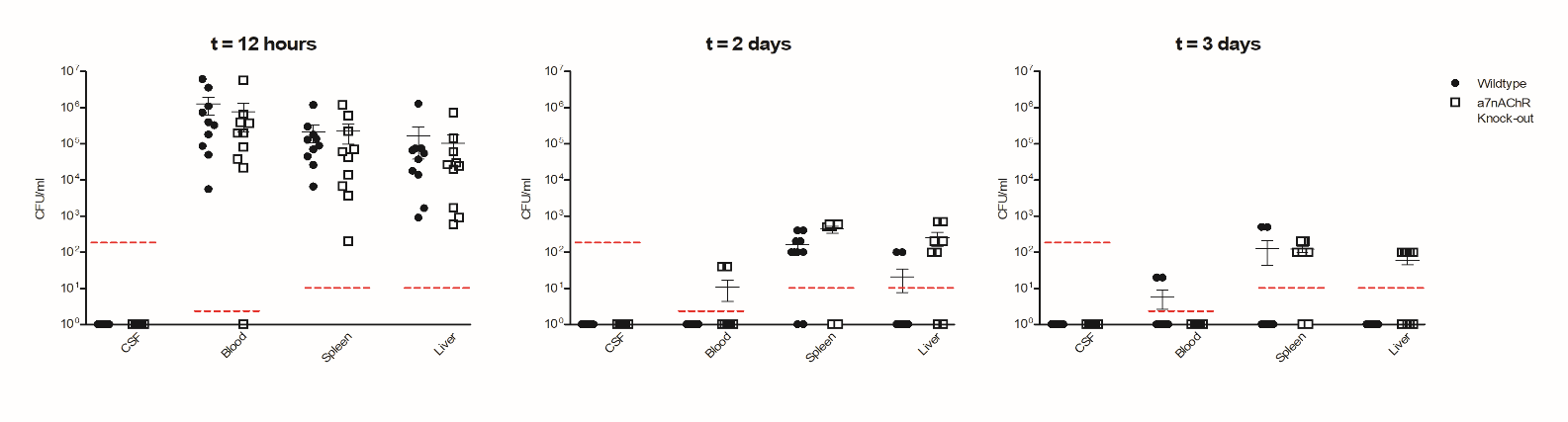


Figure S4: Bacterial outgrowth in cerebral spinal fluid (CSF), blood, spleen and liver per time point in colony-forming units per ml (CFU/ml) of all *E. coli* infected mice. Note that all mice received ceftriaxone at 12 hours and 1 day after inoculation. The red dotted line is the lower limit of detection (LLD), LLD for CSF is 2000 CFU/ml, LDD for blood is 20 CFU/ml and LDD for spleen and liver is 100 CFU/ml. Mann–Whitney U tests were performed, data represent mean + SD.

*Inflammatory mediators in brain*

Analysis of pro-inflammatory mediators (Figure S5) in infected WT mice showed decreased expression of TNF-α and IL-6 mRNA’s at day 2 and day 3 after inoculation compared to uninfected WT mice (respectively [resp.] p=0.05; p=0.0002 and p=0.003; p=0.002). In infected WT mice expression of IL-12 mRNA was increased compared to uninfected WT mice at day 2 after inoculation (p=0.01). In infected *α7nAChR^-/-^* mice expression of TNF-α mRNA was increased compared to uninfected *α7nAChR^-/-^* mice at day 2 after inoculation (p=0.01).


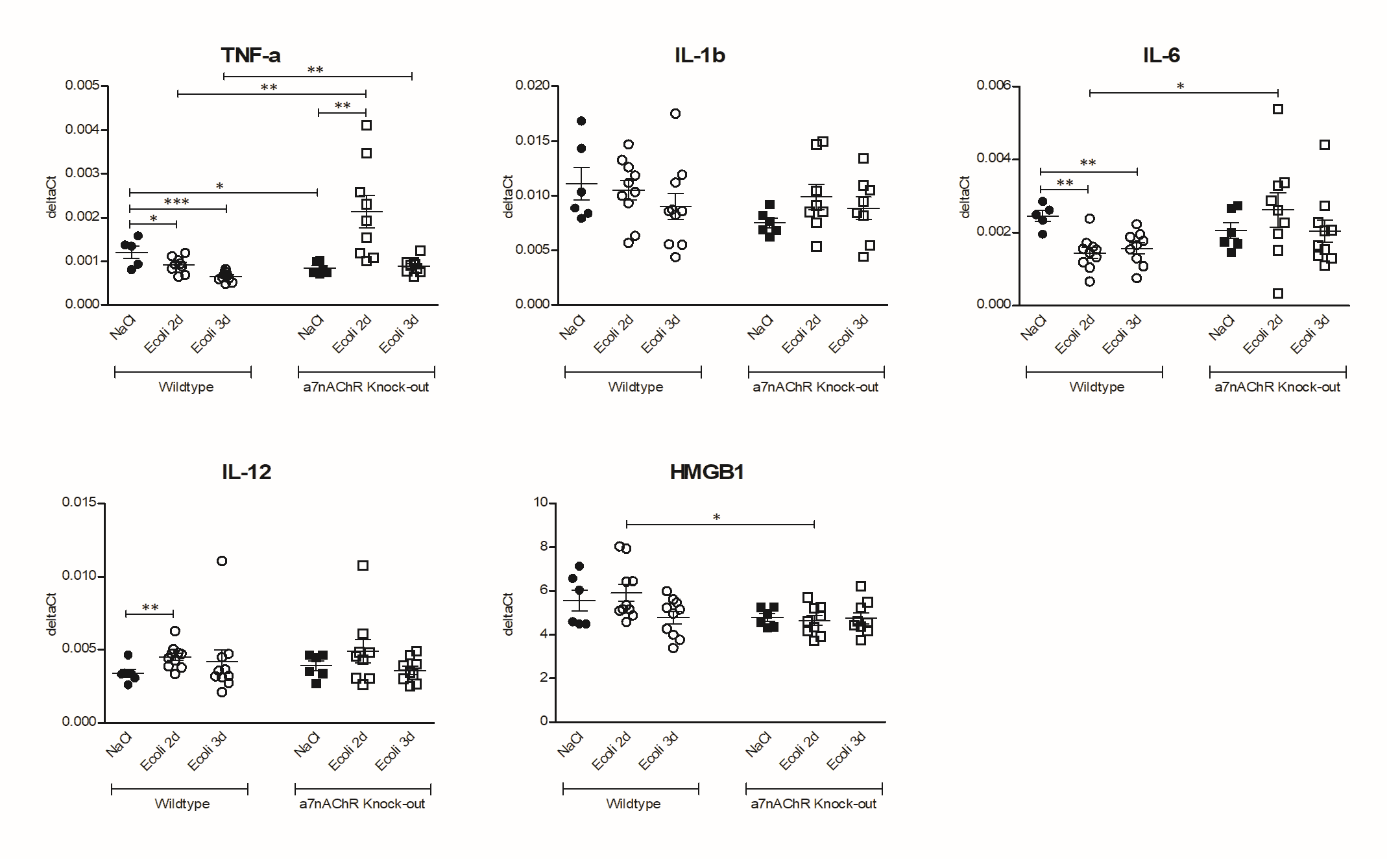


Figure S5. Expression of brain homogenate of general pro-inflammatory mediators TNF-α, IL-1β, IL-6, IL-12 and HMGB1 mRNA’s, illustrated in deltaCts. Group size varies from *n* = 5 to 10. Parametric two-way ANOVA (factors: genetics and time point) was conducted and continuous variables were tested with post-hoc Student *t*-tests for IL-1β, HMGB1. Data respresent mean + SD. Non parametrec two-way ANOVA was conducted and continuous variables were tested with post-hoc Mann–Whitney U tests for TNF-α, IL-6, IL-12. For non-parametric ANOVAs, the ranked transformation of data was performed. Data represent median + IQR. * *p* ≤ 0.05, ** *p* ≤ 0.01, *** *p* ≤ 0.001.

Analysis of immune regulators (Figure S6) in infected WT mice showed increased mRNA expression of M-CSF mRNA at day 3 after inoculation compared to uninfected WT mice (p=0.008). In infected *α7nAChR^-/-^* mice expression was increased for M-CSF and TGF-β mRNA’s at day 2 after inoculation (resp. p=0.002; p=0.004) and for M-CSF and MCP-1 mRNA’s at day 3 after inoculation (resp. p=0.02; p=0.01) compared to uninfected *α7nAChR^-/-^* mice.


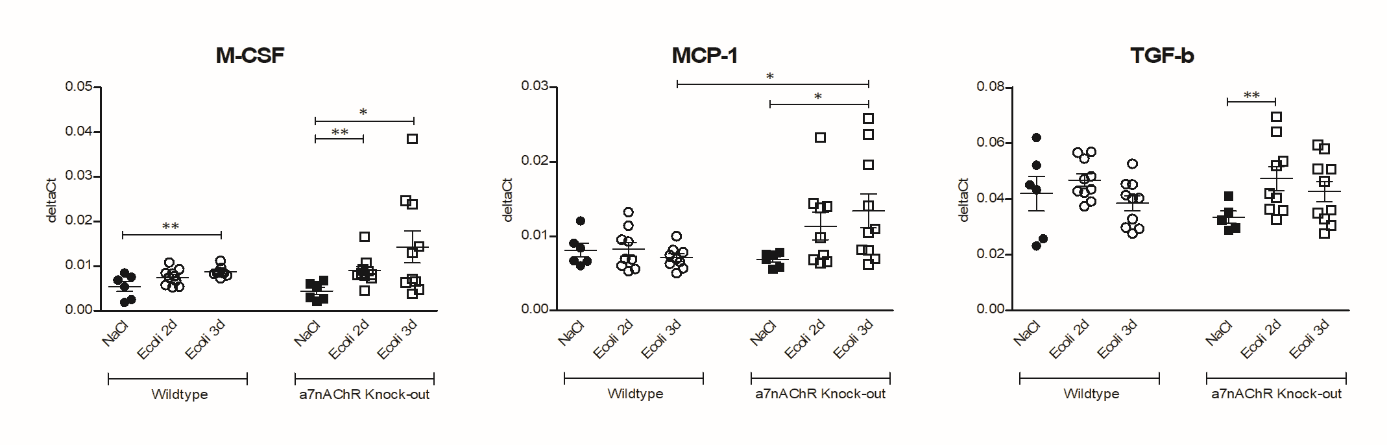


Figure S6. Expression of brain homogenate of immune regulatory mediators M-CSF, MCP-1 and TGF-β mRNA’s, illustrated in deltaCts. Group size varies from *n* = 5 to 10. Non parametrec two-way ANOVA was conducted and continuous variables were tested with post-hoc Mann–Whitney U. The ranked transformation of data was performed. Data represent median + IQR. * *p* ≤ 0.05, ** *p* ≤ 0.01.

Expression analysis of myeloid markers (Figure S7) in infected WT mice showed increased expression of Iba-1 and CD11b mRNA’s at day 2 after inoculation compared to uninfected WT mice (resp. p=0.002; p=0.001). In infected *α7nAChR^-/-^* mice there was also an increase in Iba-1 and CD11b mRNA expression at day 2 compared to uninfected *α7nAChR^-/-^* mice (resp. p=0.05; p=0.02). Comparing infected WT and *α7nAChR^-/-^* mice, the WT mice showed higher expression of CD11b mRNA at day 2 (p=0.01). There was no interaction effect for genetic background and time-point.


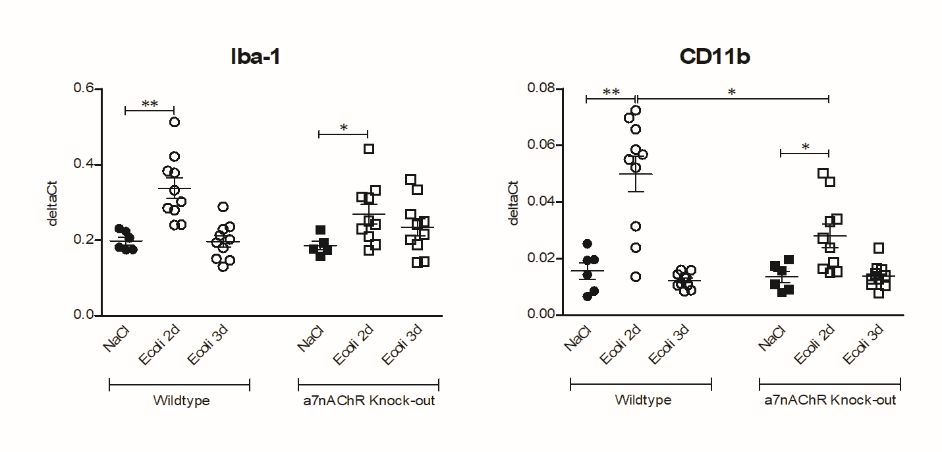


Figure S7. Expression of brain homogenate of myeloid marker Iba-1 and CD11b mRNA’s, illustrated in deltaCts. Group size varies from *n* = 5 to 10. Parametric two-way ANOVA (factors: age and time point) was conducted and continuous variables were tested with post-hoc Student *t*-tests. Data represent mean ± SD, * *p* ≤ 0.05, ** *p* ≤ 0.01.

Expression analysis of TLR cascade (Figure S8) in infected WT mice showed increased expression of MAPK1 mRNA at day 2 and day 3 after inoculation (resp. p=0.01; p=0.003) and decreased expression of SOCS1 mRNA at day 3 compared to uninfected WT mice (p=0.002). In infected *α7nAChR^-/-^* mice there was an increase in expression of TLR-2 mRNA at day 3 compared to uninfected *α7nAChR^-/-^* mice (p=0.04).


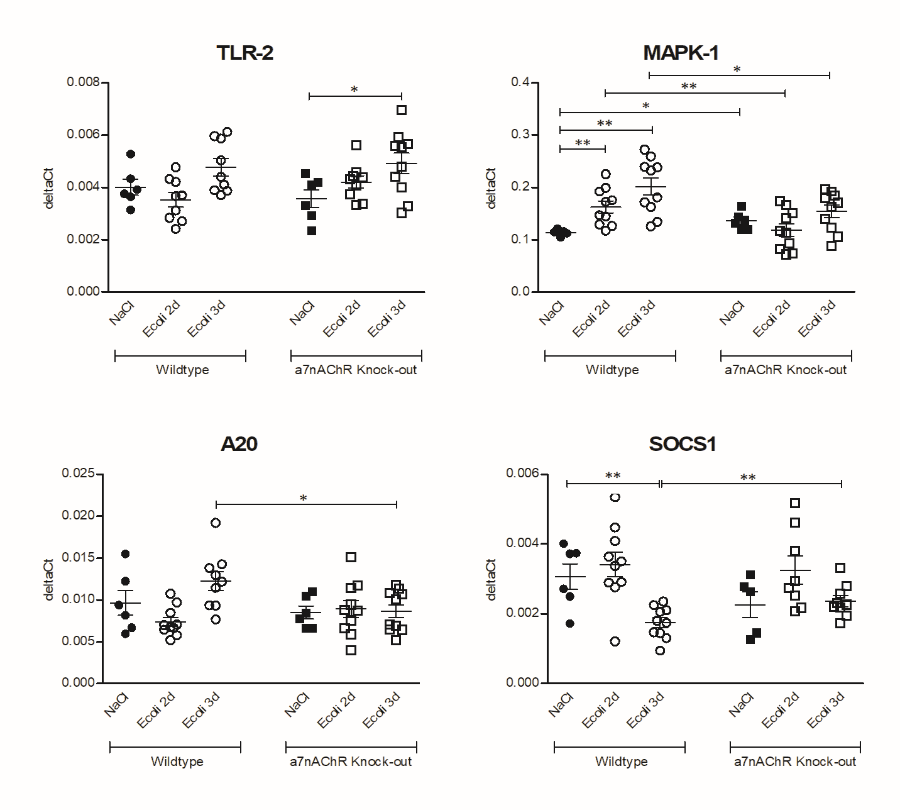


Figure S8. Expression of brain homogenate of components of the TLR signaling cascade: TLR-2, MAKP1 and A20 mRNA’s, illustrated in deltaCts. Group size varies from *n* = 5 to 10. Parametric two-way ANOVA (factors: genetics and time point) was conducted and continuous variables were tested with post-hoc Student *t*-tests for TLR-2 and A20. Data respresent mean + SD. Non parametrec two-way ANOVA was conducted and continuous variables were tested with post-hoc Mann–Whitney U tests for MAPK-1 and SOCS1. For non-parametric ANOVAs, the ranked transformation of data was performed. Data represent median + IQR. * *p* ≤ 0.05, ** *p* ≤ 0.01.

*Inflammatory mediators in spleen*

Analysis of inflammatory mediators in spleen (Figure S9) in infected WT mice showed no differences compared to uninfected WT mice for expression of TNF-α, IL-1β, IL-6, IL-12, HMGB1, M-CSF and MCP-1 mRNA’s. Infected *α7nAChR^-/-^* mice showed increased expression of HMGB1 at day 3 and MCP-1 mRNA’s at day 2 after inoculation compared to uninfected *α7nAChR^-/-^* mice (resp. p=0.005; p=0.01). There was a decrease in expression of IL-1β and IL-12 mRNA’s at day 2 (resp. p=0.009; p=0.01) and a decrease of TNF-α mRNA at day 2 and day 3 after inoculation in infected *α7nAChR^-/-^* mice compared to uninfected *α7nAChR^-/-^* mice (resp. p<0.0001; p=0.0002).


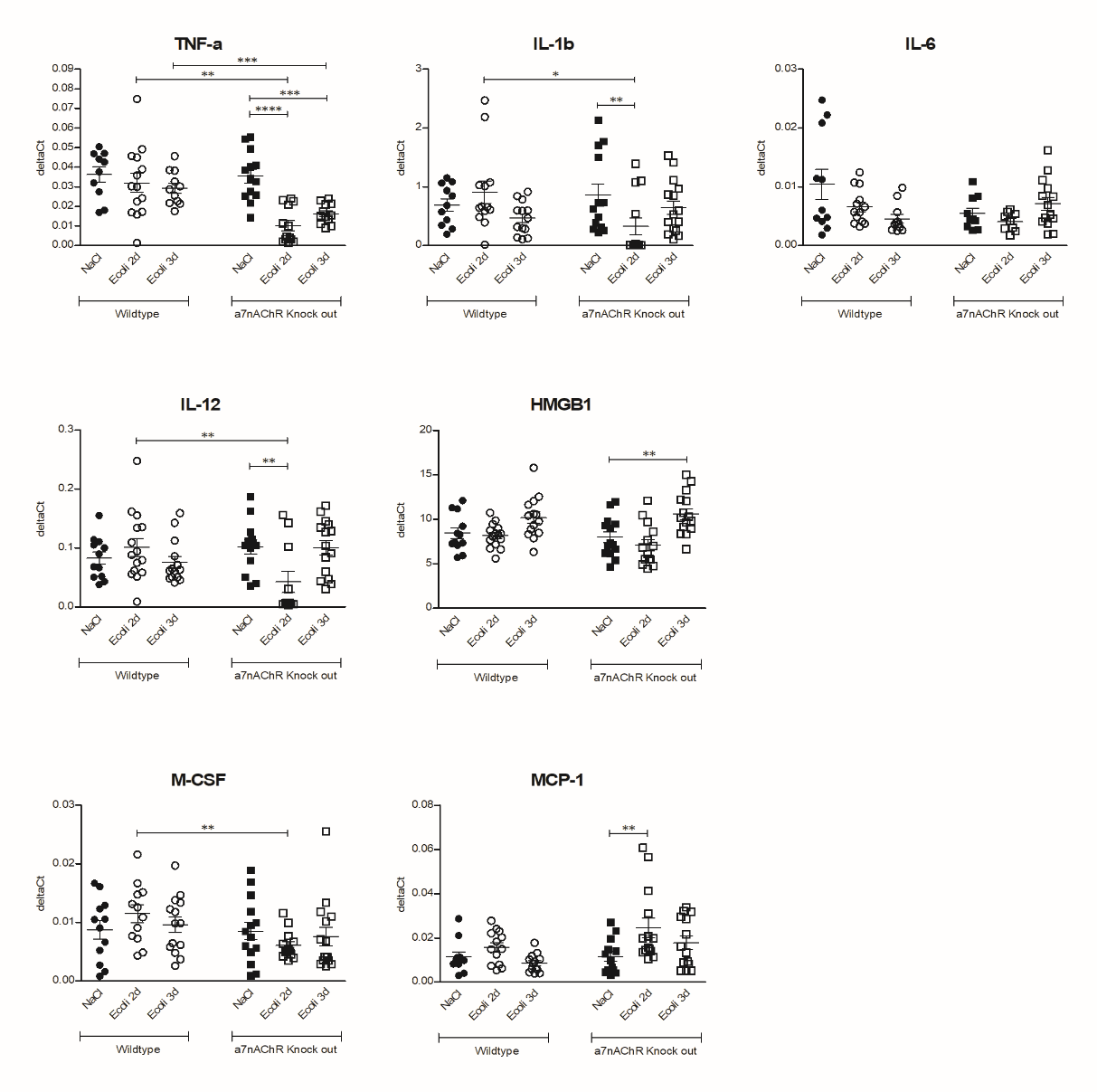


Figure S9. Expression of spleen homogenates of TNF-α, IL-1β, IL-6, IL-12, HMGB1, M-CSF and MCP-1 mRNA’s, illustrated in deltaCts. Group size varies from *n* = 10 to 15. Parametric two-way ANOVA (factors: genetics and time point) was conducted and continuous variables were tested with post-hoc Student *t*-tests for HMGB1 and M-CSF. Data respresent mean + SD. Non parametrec two-way ANOVA was conducted and continuous variables were tested with post-hoc Mann–Whitney U tests for TNF-α, IL-1β, IL-6, IL-12 and MCP-1. For non-parametric ANOVAs, the ranked transformation of data was performed. Data represent median + IQR. * *p* ≤ 0.05, ** *p* ≤ 0.01, *** *p* ≤ 0.001, **** *p* ≤ 0.0001.
